# Current state of high-fidelity multimodal monitoring in traumatic brain injury

**DOI:** 10.1007/s00701-022-05383-8

**Published:** 2022-10-19

**Authors:** Caroline Lindblad, Rahul Raj, Frederick A. Zeiler, Eric P. Thelin

**Affiliations:** 1 Department of Clinical Neuroscience, Karolinska Institutet, Stockholm, Sweden; 2 Department of Neurosurgery, Uppsala Academic Hospital, Stockholm, Sweden; 3 Department of Neurosurgery, Helsinki University Hospital and University of Helsinki, Helsinki, Finland; 4 Section of Neurosurgery, Department of Surgery, Rady Faculty of Health Sciences, University of Manitoba, Winnipeg, Canada; 5 Department of Human Anatomy and Cell Science, Rady Faculty of Health Sciences, University of Manitoba, Winnipeg, Canada; 6 Department of Biomedical Engineering, Price Faculty of Engineering, University of Manitoba, Winnipeg, Canada; 7 Division of Anaesthesia, Department of Medicine, Addenbrooke’s Hospital, University of Cambridge, Cambridge, UK; 8 Department of Neurology, Karolinska University Hospital, Stockholm, Sweden

**Keywords:** Multimodal monitoring, Traumatic brain injury, Neuro-critical care, Intracranial pressure, Biomarkers

## Abstract

**Introduction:**

Multimodality monitoring of patients with severe traumatic brain injury (TBI) is primarily performed in neuro-critical care units to prevent secondary harmful brain insults and facilitate patient recovery. Several metrics are commonly monitored using both invasive and non-invasive techniques. The latest Brain Trauma Foundation guidelines from 2016 provide recommendations and thresholds for some of these. Still, high-level evidence for several metrics and thresholds is lacking.

**Methods:**

Regarding invasive brain monitoring, intracranial pressure (ICP) forms the cornerstone, and pressures above 22 mmHg should be avoided. From ICP, cerebral perfusion pressure (CPP) (mean arterial pressure (MAP)–ICP) and pressure reactivity index (PRx) (a correlation between slow waves MAP and ICP as a surrogate for cerebrovascular reactivity) may be derived. In terms of regional monitoring, partial brain tissue oxygen pressure (PbtO_2_) is commonly used, and phase 3 studies are currently ongoing to determine its added effect to outcome together with ICP monitoring. Cerebral microdialysis (CMD) is another regional invasive modality to measure substances in the brain extracellular fluid. International consortiums have suggested thresholds and management strategies, in spite of lacking high-level evidence. Although invasive monitoring is generally safe, iatrogenic hemorrhages are reported in about 10% of cases, but these probably do not significantly affect long-term outcome. Non-invasive monitoring is relatively recent in the field of TBI care, and research is usually from single-center retrospective experiences. Near-infrared spectrometry (NIRS) measuring regional tissue saturation has been shown to be associated with outcome. Transcranial doppler (TCD) has several tentative utilities in TBI like measuring ICP and detecting vasospasm. Furthermore, serial sampling of biomarkers of brain injury in the blood can be used to detect secondary brain injury development.

**Conclusions:**

In multimodal monitoring, the most important aspect is data interpretation, which requires knowledge of each metric’s strengths and limitations. Combinations of several modalities might make it possible to discern specific pathologic states suitable for treatment. However, the cost–benefit should be considered as the incremental benefit of adding several metrics has a low level of evidence, thus warranting additional research.

## Introduction

Traumatic brain injury (TBI) is one of the most common causes of death and acquired disability worldwide, with the greatest burden suffered by low- and middle-income countries [22]. TBI has been stated to be the most complex and heterogeneous disease in the most complex organ [59], which is one of the main reasons that has been suggested why so far all pharmaceutical trials targeting the underlying pathophysiology in TBI have failed to demonstrate efficacy [10, 13, 24]. Presumably, one of the best ways to discern specific pathological states which could be amenable to treatment, and hence increase the possibilities of success of future trials, is through improved monitoring [51, 61].

In severe TBI, where the patient is unconscious at the scene of injury or at admission to the hospital (commonly assessed using the Glasgow Coma Scale (GCS) with a score < 9) [50], admission to specialized neuro-critical care units (NCCU) using standardized management protocols is recommended [26]. The main goal of these is to reduce the burden of secondary brain insults in order to optimize recovery. To facilitate this, institutions like the Brain Trauma Foundation (BTF, the fourth iteration of guidelines published in 2017) [5] and the Seattle International Severe Traumatic Brain Injury Consensus Conference (SIBICC, from 2020) [17] provide a framework of recommendations for different monitoring modalities, the level of clinical evidence as well as stipulating relevant thresholds and ranges.

Despite the lack of higher levels of evidence of efficacy, several monitoring modalities, both invasive and non-invasive, often in combination, are used in specific centers today. This brief review will cover the most commonly used modalities, the current state of ongoing trials, and recommendations on how to optimize multimodal monitoring in severe TBI.

## Invasive monitoring

### Intracranial pressure

One of the cornerstones in neurosurgery involves the Monro–Kellie doctrine, entailing that for an expanding intracranial mass, there will be a compensation (initially through an intracranial decrease of cerebrospinal fluid (CSF) and intracranial venous blood) [29]. At the outset, the brain has a considerable elastance in order to accompany volumetric increases, but as an intracranial mass increases, this compliance decreases which results in an incremental increase of ICP. If left untreated, these increases will result in cerebral herniation, both subfalcine and uncal, where the latter may result in circulatory collapse. The gold standard technique to measure ICP is through a closed external ventricular drain (EVD); however, today, intraparenchymal monitoring devices (commonly abbreviated ICPM, “ICP monitor”) are commonly used. Both techniques have pros and cons, with the EVD being cheaper and has a possibility to drain CSF in order to treat elevated ICP (though it then cannot accurately measure ICP), though it carries a slightly higher risk profile [58]. However, an ICPM is easier to insert than an EVD [49], which is done bedside in some centers. While the initial EVDs could not drain CSF and measure ICP at the same time, novel types of pressure sensors in EVDs or CSF pumps make this possible [54].

The BTF guidelines provide level II B evidence suggesting that management of severe TBI patients using information from ICP monitoring is recommended to reduce in-hospital and 2-week post-injury mortality, and ICP levels below 22 mmHg are associated with a favorable outcome [5, 45]. While a meta-analysis from 2010 shows that both mortality and functional outcome improve [46], increased ICP is primarily associated with an increased risk of death following TBI [45]. The ICP monitoring paradigm was challenged in 2012 when Randall Chesnut and coworkers published a randomized trial of ICP monitoring in TBI and could show that the ICP-monitored arm had neither a better short- nor long-term outcome vs a group where clinicians treated ICP according to clinical and radiological findings (the Benchmark Evidence from South American Trials: Treatment of Intracranial Pressure (BEST:TRIP trial)) [8]. However, the true take-home message from BEST:TRIP, which is also suggested by Chesnut [7], is not a poor efficacy of ICP monitoring, but that aggressive ICP management works better if you know what you are doing than if you are guessing, as monitoring halved the number of ICP lowering treatments administered per patient. Additionally, the failure of BEST:TRIP to show efficacy with only ICP monitoring suggests that additional monitoring modalities may be necessary in order to improve outcome. The results of BEST:TRIP have also been challenged by several experts in the field for its issues with validity [21, 42] and show on the complexity when extrapolating results from low- and middle-income countries’ health care systems to those in high-income countries.

### Cerebral perfusion pressure (CPP) and pressure reactivity index (PRx)

From ICP, other metrics can be derived, such as CPP (mean arterial pressure (MAP)–ICP) and PRx (a correlation between slow waves of MAP and ICP) [9]. The CPP is the net pressure gradient resulting in cerebral perfusion and a common surrogate for the cerebral blood flow in the brain and is (apart from lowering ICP) commonly managed by increasing MAP. While different schools exist on how to manage CPP, with the Lund concept promoting a more careful approach (with a range of 50–70 mmHg) [15], the range of 60–70 mmHg is now recommended in the 2017 BTF and SIBICC guidelines where levels above 70 mmHg have been stated to be counterproductive, with a level II evidence grade [5, 17] (though it should be stressed that the location of the zero level of MAP and ICP is essential [39]). They do also state that autoregulatory capacity of the patients should be taken into consideration when deciding on optimal CPP for the patient. PRx is a surrogate for cerebrovascular autoregulation [9], measured using a range from 0 indicating perfect autoregulation and 1 which is complete inability to autoregulate blood flow. BTF states that a PRx > 0.25 has been shown to be associated with mortality and > 0.05 with unfavorable outcome (level II evidence) [5, 45]. Similarly, recent work has highlighted that the majority of patients spend over 50% of any given day in the ICU with impaired cerebral autoregulation [60], with impaired autoregulation appearing to dominate the landscape of impaired cerebral physiology in moderate/severe TBI during periods of controlled ICP and CPP [1]. Further to this, data supports the relative treatment independence of cerebral autoregulation to current guideline-based therapeutic approaches in TBI care, leaving us to focus on strategies aimed at finding the “least worst” cerebral autoregulatory state for a given patient [13, 14, 60].

Joint work from Sweden and the UK showed that patients with a negative PRx correlation should be targeted with CPP strategies, while those with a positive correlation should have an ICP lowering focus in order to improve outcome [19]; thus, more modalities might facilitate management strategies. Furthering this, the CPP where the PRx is the lowest is referred to as the “optimal CPP” [2, 47], and in the recently published study CPPOpt Guided Therapy: Assessment of Target Effectiveness (COGITATE), a prospective study where *n* = 30 was randomized to CPP 60–70 mmHg and *n* = 30 to individualized CPPopt targets; the authors could show that CPPopt management was safe [48]. Larger trials are warranted to show clinical efficacy.

### Partial brain tissue oxygen pressure (PbtO2)

**Fig. 1:**
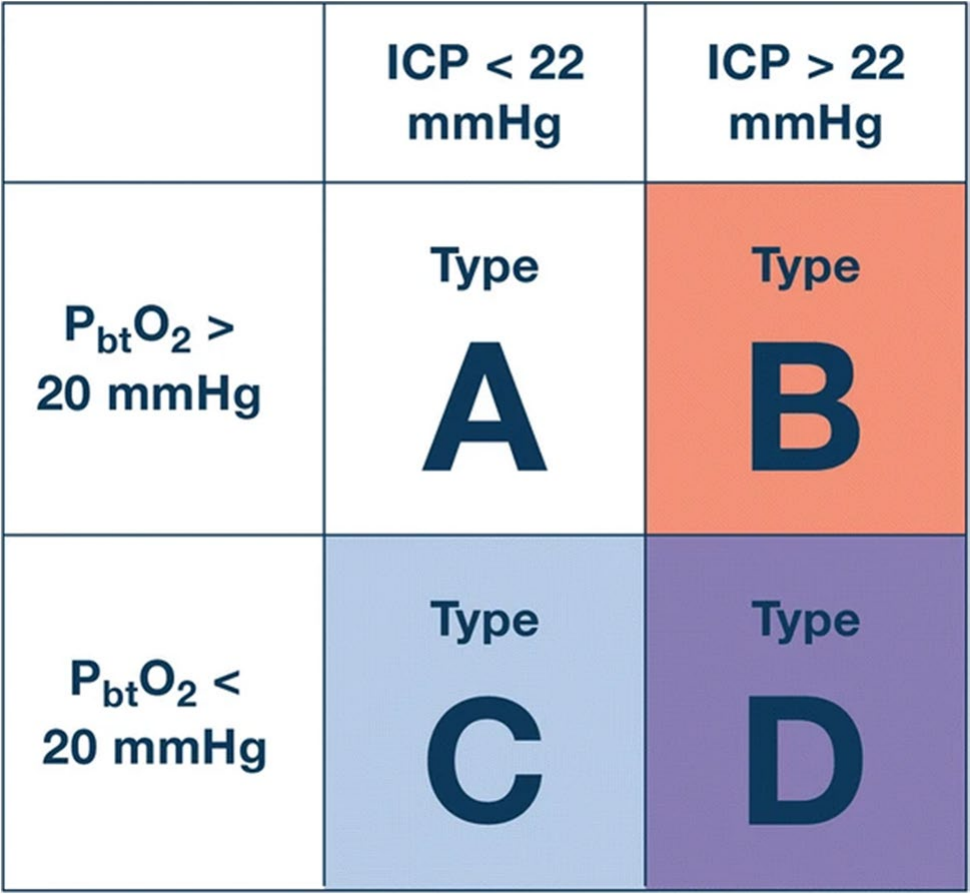
Example of different definitions of different TBI phenotypes based on multimodal monitoring approach using ICP and PbtO_2_. ICP intracranial pressure, PbtO_2_ partial brain tissue oxygen pressure. This figure was originally published by Chesnut et al. [6] © 2020 The Authors. Published by Springer-Nature. Open Access under a CC–BY license

Regional cerebral oxygenation may be measured using invasive catheters using different techniques to measure the PbtO_2_ (sometimes referred to as PbrO_2_). The BTF states level II evidence for an increased mortality risk of PbtO_2_ < 29 mmHg and level III evidence for unfavorable outcome at a range below 15–20 mmHg [5, 18]. Several PbtO_2_ monitors on the market also monitor cerebral temperature, which in itself is important to monitor as it differs from core temperature monitoring [27], but has also been shown to influence intracranial dynamics [4]. PbtO_2_ is becoming the second most common monitored metric apart from ICP, but the evidence on what it adds in addition to ICP monitoring is scarce. However, in 2017, the Brain Tissue Oxygen Monitoring and Management in Severe Traumatic Brain Injury (BOOST-2) trial was published which showed adequate safety with PbtO_2_ monitoring as well as a trend towards improved outcome in the PbtO_2_ + ICP cohort vs the cohort only monitored using ICP [33]. These results have led to fine-tuning of treatment algorithms as well as a power calculation to perform the North American phase III trial BOOST-3 which is currently underway [3] and aims to provide level 1 evidence of outcome efficacy with PbtO_2_ + ICP monitoring. Other similar trials are the French OXY-TC trial [34] and the Brain Oxygen Neuromonitoring in Australia and New Zealand Assessment (BONANZA) trial (ACTRN12619001328167), which both use specified tiered algorithms in order to target predefined conditions depending on the information from ICP and PbtO_2_ monitoring to see if these are better than ICP monitoring in isolation. Similarly, SIBICC guidelines already suggest suitable treatment recommendations based on available evidence when combining ICP and PbtO_2_ [6] by defining specific TBI phenotypes based on multimodality monitoring (Fig. 1).

### Cerebral microdialysis (CMD)

CMD consists of a catheter with a semipermeable membrane which is inserted into the brain parenchyma, connected to a pump through which substances flow from the brain extracellular fluid (ECF) into tubing through osmosis [51]. In severe TBI, CMD is primarily used to monitor metabolites at the bedside using point-of-care devices [20]. While CMD measuring glutamate (increasing levels were associated with an increased mortality vs lower levels) is stated as level 3 evidence in the BTF [5], there are several similar retrospective studies for thresholds for glucose, lactate, pyruvate as well as the lactate:pyruvate ratio (LPR) [63]. Studies from the University of Cambridge, UK have shown that deranged cerebral metabolism with LPR > 25 is associated with outcome [56] and that when the brain suffers from high LPR, it also has low ECF glucose, low PbtO_2_, and an impaired PRx [16]. Similarly, studies from Sweden have combined LPR with glucose and pyruvate levels in order to create a state of “ischemia” (high lactate and LPR with low pyruvate) and “mitochondrial dysfunction” (high lactate and LPR despite normal pyruvate levels) [31] and seen that these are common in different TBI pathologies [32].

### Combinations of several monitoring modalities

Apart from the above-mentioned studies, there are regional centers that have used ICP, PbtO_2_, and CMD monitoring in combination for a period of time and gained experience from these. One such center is Addenbrooke’s Hospital, Cambridge, UK which published recommendations for thresholds for these metrics in 2017 (Table 1) [28]. Albeit not based on other than level 3 evidence, and not specifying what each added modality provides for additional monitoring or predictive capability, they do provide guidance for centers using these monitoring modalities in combination. Recently, the group published a paper where an LPR-driven algorithm (if LPR > 25) was used with a tiered therapy escalating from initially correcting ICP if above 20 mmHg (intracranial hypertension), then adjusting CPP if PbtO_2_ was < 20 mmHg (oxygen delivery failure), followed by increasing serum glucose to 10 mmol/l if brain glucose < 1.0 mmol/l (neuroglycopenia) [23]. If nothing corrected the LPR, the patient was deemed to suffer from mitochondrial dysfunction. They then mapped exactly which neurometabolic state (NMS) the patient was in the first 2 weeks following injury. Apart from a normal LPR, the two most common NMS were mitochondrial dysfunction and neuroglycopenia, while intracranial hypertension and PbtO_2_ issues were rare [23]. By dividing patients in different NMS, it was also possible to specifically target the group with a mitochondrial dysfunction. Approaches such as this will presumably allow for better monitoring in larger trials to target specific patient groups in the heterogenic landscape of TBI, which could tailor treatments specifically to each patient.

**Table 1:** Recommendations for multimodality monitoring using ICP, PbtO_2_, and CMD monitoring. Derived and modified from [28]. ICP intracranial pressure, PbtO_2_ partial brain tissue oxygen pressure, CMD cerebral microdialysis, PRx pressure reactivity index, CPPopt optimal cerebral perfusion pressure

Modality	Normal	Desirable	Injury threshold
ICP	~ 10 mmHg	< 20 mmHg	> 22–25 mmHg
PRx for CPPopt	< 0	< 0.05	> 0.25
PbtO_2_	~ 30 mmHg	20–25 mmHg	< 15 mmHg
Lactate:pyruvate ratio	< 25	< 25	> 25
Brain glucose	1–2 mmol/L	< 0.8 mmol/L	< 0.5 mmol/L

### Risks and difficulties with invasive intracerebral monitoring

**Fig. 2:**
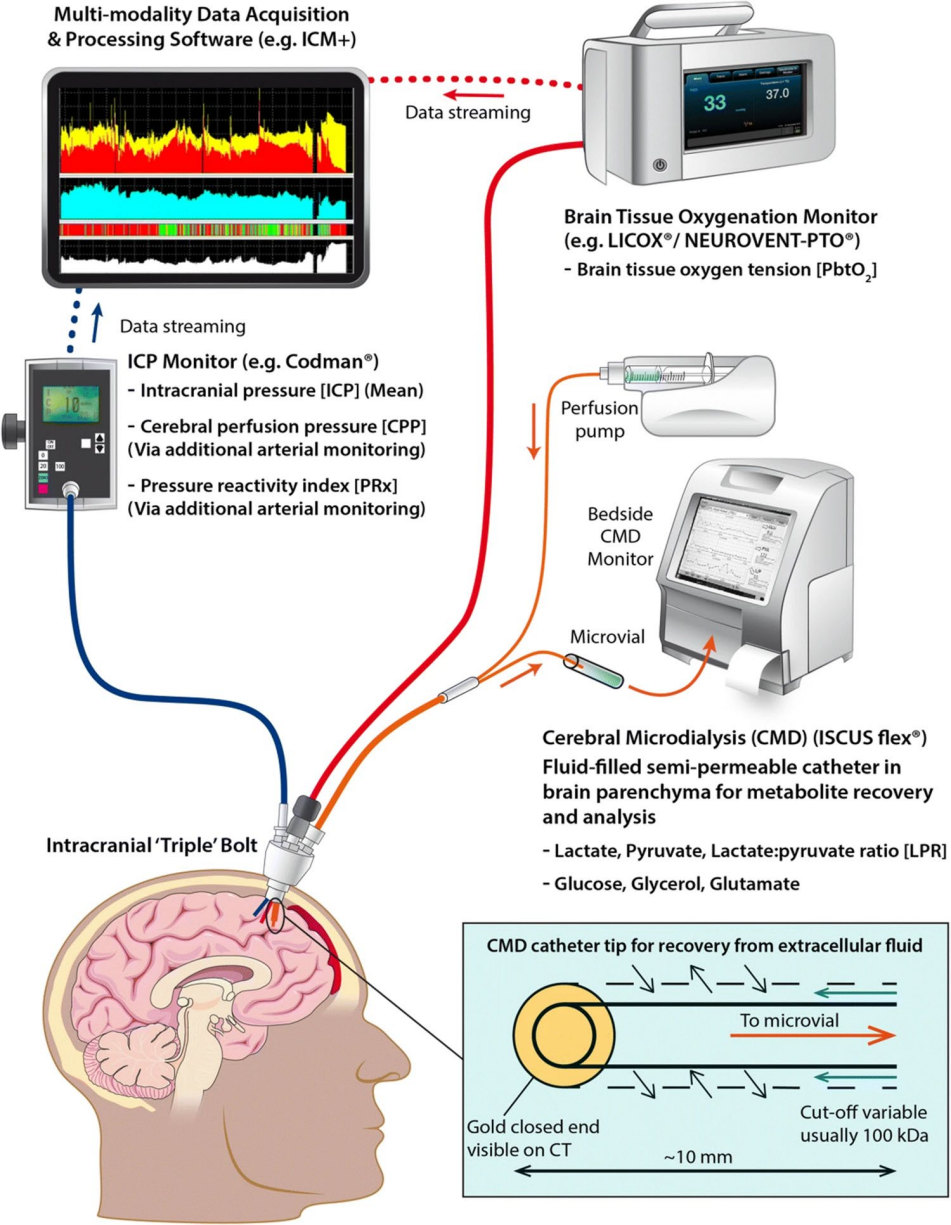
Setup of invasive multimodal monitoring combining ICP, PbtO_2_, and CMD monitoring. ICP intracranial pressure, PbtO_2_ partial brain tissue oxygen pressure, CMD cerebral microdialysis, PRx pressure reactivity index, CPP cerebral perfusion pressure. This figure was originally published by Khellaf et al. [24] © 2019 The Authors. Published by Springer-Nature. Open Access under a CC–BY license

Invasive monitoring is not without risks. Tavakoli and colleagues in a review from 2017 reported on EVD associated infection rates that are around 10%, about 1% for ICPM, and hemorrhages (albeit subclinical in many cases) to be around 30% in some studies following EVD insertion (about 1% for ICPMs) [49]. In a recent study by Pease et al. [35], they found that in *n* = 599 severe TBI patients, there were 12% hemorrhages and 7% CSF verified infections caused by invasive monitoring. However, while patients with complications had a longer hospital stay, there were no differences in outcome following 6 months [35]. Many catheters are also inserted beyond manufacturers recommendations, commonly 20 mm in right frontal white matter, but instead ending up with a range of between 9 and 42 mm [43]. Because of this, bolts and other intracranial access devices are commonly used which prevent this by better fixation of intracranial monitors. An example of this setup is shown (Fig. 2).

Another aspect of invasive monitoring involves the limitations of a too small area that is monitored. It has been shown that when monitoring patients with three different CMD catheters, depending on the location of the catheter and proximity to intracranial lesions, there is a notable difference in lactate:pyruvate and glucose levels [11, 30]. A similar issue is seen with PbtO_2_ monitors showing lower levels when placed in abnormal tissue [36]. While this could be desirable as a border zone that is more susceptible to injury could be monitored and more rapidly indicate deterioration, in many cases, this increases the complexity of multimodal monitoring which may further prevent a more mainstream use. Therefore, while some centers are targeting certain areas for regional monitoring, many are instead aiming for healthy tissue in order to assess a more “global” measurement (also recommended by the most recent consensus for CMD monitoring [20]).

## Non-invasive monitoring

While invasive monitoring techniques are gold standard within the TBI field, non-invasive monitoring techniques exist. They are commonly cheaper, less risky, and offer an easier approach compared to invasive techniques, and as research progresses, they might replace some of the invasive techniques commonly used today.

### Near-infrared spectroscopy (NIRS)

While not supported by the BTF guidelines, the NIRS technique has been used since the 1970s in order to measure oxygenated hemoglobin. Regional hypoxia measured using continuous cranial NIRS has been shown to be correlated with unfavorable outcome, increased ICP and low PbtO_2_, as shown in a systematic review from 2020 [25]. However, NIRS regional saturation has been shown to be associated with extracranial noise [44], which has somewhat hampered its more widespread clinical utility.

### Transcranial doppler (TCD)

Currently, the most recent BTF guidelines state that there is insufficient evidence to recommend TCD monitoring [5]. However, TCD monitoring has many different utilities following TBI and may be used to estimate flow velocity in vessels as to asses, e.g., vasospasm [64] and ICP [40]. By using ultrasound technology, the method is relatively harmless. Additionally, in many centers where invasive ICP monitoring is not available, optic nerve sheet diameter (ONSD) assessments may also work as a surrogate for ICP [41]. A drawback with TCD is that it is intermittent in nature and very user-dependent; however, new robotic TCDs are being introduced and may facilitate this type of monitoring, as a key advantage then becomes the opportunity for user-independent longitudinal assessments of each patient [62].

### Serial sampling of protein biomarker of brain injury

Protein biomarkers of tissue fate are used in several aspects of medicine today and have been introduced in TBI management as well [53]. While most commonly used to screen for lesions in mild TBI patients in order to rule out the need for a computerized tomography (CT) scan [57], serial sampling of protein biomarkers may be performed in order to monitor for progression of lesions or development of new injuries [37]. The most studied blood biomarker is the primarily astrocytic protein S100B. Other proteins that have been studied in this setting include neuron-specific enolase (NSE), glial fibrillary acidic protein (GFAP), tau, neurofilament-light (NfL), and ubiquitin carboxy-terminal hydrolase L1 (UCH-L1) [55]. Even relatively modest increases of S100B (> 0.05 µg/L) if sampled every 12 h have a robust sensitivity and specificity in order to detect lesions seen on imaging [52]. Currently, blood biomarkers are not a part of the BTF guidelines [5].

## Other issues related to neuromonitoring

### Costs

The expenses associated with neuromonitoring vary to some extent from center to center. However, factoring in consumables only, an ICPM costs about 500 USD for a week (an EVD about 200–300 USD), while PbtO_2_ costs about 1000 USD and CMD about 700 USD. Non-invasive monitoring is cheap, and for techniques like the TCD, the amount of consumables is negligible, while NIRS sensors cost about 150 USD (for two bilateral sensors). Serial sampling of 2 tests of S100B per day for a week will cost about 300 USD. It should be acknowledged that it is difficult to perform adequate cost:benefit analyses in this area due to the heterogeneity of severe TBI and the lack of larger trials. Though, in order to justify costs for using several monitoring modalities, especially in cost-sensitive settings such as low- and middle-income countries, studies clearly showing added benefit are warranted.

### Interpretation of monitoring data

While guidelines such as those provided by BTF and the international consensus meeting for CMD monitoring provide thresholds for different parameters and metabolites [5, 20], the dynamics of multimodal monitoring are usually more complex. More often trends and trajectories are more relevant than absolute numbers, which are difficult to adequately describe in guidelines. Additionally, it is important to be able to trust the sensors, and the location of many sensors, such as inside a cerebral contusion or close to the dura, could make CMD and PbtO_2_ measurements unreliable and hence difficult to guide therapy. Therefore, multimodality monitoring relies a lot on quality control and different indirect tests, like increasing the flow of oxygen (FiO_2_) to 100% in order to see a response on the PbtO_2_, to trust your monitors. In the future, artificial intelligence (AI) and different machine learning approaches may facilitate the interpretation of multimodal monitoring signals. Currently, this has been shown to improve dynamic predictions in patients monitored using ICP [38] but is still in its infancy when it comes to incorporating several modalities in real time [12].

Most importantly, however, is that just inserting fancy and expensive monitoring equipment will not improve the outcome and management of TBI patients. Instead, this will come to the staff and physicians in the NCCU that interprets and acts on the signals provided to promptly guide diagnostics and potential changes in management, and educational and training efforts are necessary to uphold an adequate level of competence.

Then, what are the best combinations of modalities? To combine invasive (e.g., ICP) and non-invasive (e.g., NIRS)? To have one regional (e.g., CMD or PbtO_2_) and one global (e.g., ICP or serial S100B sampling)? To have one continuous (e.g., ICP or PbtO_2_) and one intermittent (e.g., TCD)? As there is no robust evidence supporting anything else than ICP monitoring, and even that could be debated, the addition of several monitors comes down to regional/center preference, conditions, and infrastructure. Commonly, if a center is used to working with a set of modalities that is perhaps not as widespread globally, like the more dated jugular bulb oximetry instead of PbtO_2_ monitoring, if the experience exists in the organization on how to act and manage the information this provides, it might be better than other combinations for that particular center.

## Summary

While invasive ICP monitoring has been the established gold standard in TBI monitoring for some time, it is not able to detect smaller lesions, e.g., ischemic injury, early. This is one of the reasons why multimodal monitoring approaches using PbtO_2_ and/or CMD monitoring are being used more and more in centers worldwide. As a general rule, and what was clearly shown by the BEST-TRIP trial, more monitoring will make us know, so we do not have to guess who to treat and when. Non-invasive techniques like NIRS and TCD monitoring may to some extent replace some invasive monitoring, but additional studies are warranted.

Currently, the evidence for the added benefit using several monitoring modalities is low. But several studies are ongoing to establish the role of, e.g., PbtO_2_ + ICP. These types of studies will be able to discern specific pathological states in TBI susceptible for different types of treatment. While there are no pharmacologically approved therapies targeting the underlying pathophysiology of TBI, multimodal monitoring will be one of the solutions that provide better granularity on secondary injury processes which can be targeted with more sophisticated therapies in the future.

## References

[1] Akerlund CA, Donnelly J, Zeiler FA, Helbok R, Holst A, Cabeleira M, Guiza F, Meyfroidt G, Czosnyka M, Smielewski P, Stocchetti N, Ercole A, Nelson DW, Participants C-THRIS-S, Investigators (2020). Impact of duration and magnitude of raised intracranial pressure on outcome after severe traumatic brain injury: a CENTER-TBI high-resolution group study. PLoS One.

[2] Aries MJ, Czosnyka M, Budohoski KP, Steiner LA, Lavinio A, Kolias AG, Hutchinson PJ, Brady KM, Menon DK, Pickard JD, Smielewski P (2012). Continuous determination of optimal cerebral perfusion pressure in traumatic brain injury. Crit Care Med.

[3] Bernard F, Barsan W, Diaz-Arrastia R, Merck LH, Yeatts S, Shutter LA (2022). Brain Oxygen Optimization in Severe Traumatic Brain Injury (BOOST-3): a multicentre, randomised, blinded-endpoint, comparative effectiveness study of brain tissue oxygen and intracranial pressure monitoring versus intracranial pressure alone. BMJ Open.

[4] Birg T, Ortolano F, Wiegers EJA, Smielewski P, Savchenko Y, Ianosi BA, Helbok R, Rossi S, Carbonara M, Zoerle T, Stocchetti N, Investigators C-T, Participants,  (2021). Brain temperature influences intracranial pressure and cerebral perfusion pressure after traumatic brain injury: a CENTER-TBI study. Neurocrit Care.

[5] Carney N, Totten AM, O’Reilly C, Ullman JS, Hawryluk GW, Bell MJ, Bratton SL, Chesnut R, Harris OA, Kissoon N, Rubiano AM, Shutter L, Tasker RC, Vavilala MS, Wilberger J, Wright DW, Ghajar J (2017). Guidelines for the management of severe traumatic brain injury, Fourth Edition. Neurosurgery.

[6] Chesnut R, Aguilera S, Buki A, Bulger E, Citerio G, Cooper DJ, Arrastia RD, Diringer M, Figaji A, Gao G, Geocadin R, Ghajar J, Harris O, Hoffer A, Hutchinson P, Joseph M, Kitagawa R, Manley G, Mayer S, Menon DK, Meyfroidt G, Michael DB, Oddo M, Okonkwo D, Patel M, Robertson C, Rosenfeld JV, Rubiano AM, Sahuquillo J, Servadei F, Shutter L, Stein D, Stocchetti N, Taccone FS, Timmons S, Tsai E, Ullman JS, Vespa P, Videtta W, Wright DW, Zammit C, Hawryluk GWJ (2020). A management algorithm for adult patients with both brain oxygen and intracranial pressure monitoring: the Seattle International Severe Traumatic Brain Injury Consensus Conference (SIBICC). Intensive Care Med.

[7] Chesnut RM (2013). Intracranial pressure monitoring: headstone or a new head start. The BEST TRIP trial in perspective. Intensive Care Med.

[8] Chesnut RM, Temkin N, Carney N, Dikmen S, Rondina C, Videtta W, Petroni G, Lujan S, Pridgeon J, Barber J, Machamer J, Chaddock K, Celix JM, Cherner M, Hendrix T (2012). A trial of intracranial-pressure monitoring in traumatic brain injury. N Engl J Med.

[9] Czosnyka M, Smielewski P, Kirkpatrick P, Laing RJ, Menon D, Pickard JD (1997). Continuous assessment of the cerebral vasomotor reactivity in head injury. Neurosurgery.

[10] Donnelly J, Czosnyka M, Adams H, Cardim D, Kolias AG, Zeiler FA, Lavinio A, Aries M, Robba C, Smielewski P, Hutchinson PJA, Menon DK, Pickard JD, Budohoski KP (2019). Twenty-five years of intracranial pressure monitoring after severe traumatic brain injury: a retrospective, single-center analysis. Neurosurgery.

[11] Engstrom M, Polito A, Reinstrup P, Romner B, Ryding E, Ungerstedt U, Nordstrom CH (2005). Intracerebral microdialysis in severe brain trauma: the importance of catheter location. J Neurosurg.

[12] Foreman B, Lissak IA, Kamireddi N, Moberg D, Rosenthal ES (2021). Challenges and opportunities in multimodal monitoring and data analytics in traumatic brain injury. Curr Neurol Neurosci Rep.

[13] Froese L, Batson C, Gomez A, Dian J, Zeiler FA (2021). The limited impact of current therapeutic interventions on cerebrovascular reactivity in traumatic brain injury: a narrative overview. Neurocrit Care.

[14] Froese L, Dian J, Batson C, Gomez A, Unger B, Zeiler FA (2020). Cerebrovascular response to propofol, fentanyl, and midazolam in moderate/severe traumatic brain injury: a scoping systematic review of the human and animal literature. Neurotrauma Rep.

[15] Grande PO (2017). Critical evaluation of the Lund concept for treatment of severe traumatic head injury, 25 years after its introduction. Front Neurol.

[16] Guilfoyle MR, Helmy A, Donnelly J, Stovell MG, Timofeev I, Pickard JD, Czosnyka M, Smielewski P, Menon DK, Carpenter KLH, Hutchinson PJ (2021). Characterising the dynamics of cerebral metabolic dysfunction following traumatic brain injury: a microdialysis study in 619 patients. PLoS ONE.

[17] Hawryluk GWJ, Aguilera S, Buki A, Bulger E, Citerio G, Cooper DJ, Arrastia RD, Diringer M, Figaji A, Gao G, Geocadin R, Ghajar J, Harris O, Hoffer A, Hutchinson P, Joseph M, Kitagawa R, Manley G, Mayer S, Menon DK, Meyfroidt G, Michael DB, Oddo M, Okonkwo D, Patel M, Robertson C, Rosenfeld JV, Rubiano AM, Sahuquillo J, Servadei F, Shutter L, Stein D, Stocchetti N, Taccone FS, Timmons S, Tsai E, Ullman JS, Vespa P, Videtta W, Wright DW, Zammit C, Chesnut RM (2019). A management algorithm for patients with intracranial pressure monitoring: the Seattle International Severe Traumatic Brain Injury Consensus Conference (SIBICC). Intensive Care Med.

[18] Hays LMC, Udy A, Adamides AA, Anstey JR, Bailey M, Bellapart J, Byrne K, Cheng A, Jamie Cooper D, Drummond KJ, Haenggi M, Jakob SM, Higgins AM, Lewis PM, Hunn MK, McNamara R, Menon DK, Murray L, Reddi B, Trapani T, Vallance S, Young PJ, Diaz-Arrastia R, Shutter L, Murray PT, Curley GF, Nichol A (2022). Effects of brain tissue oxygen (PbtO2) guided management on patient outcomes following severe traumatic brain injury: a systematic review and meta-analysis. J Clin Neurosci.

[19] Howells T, Elf K, Jones PA, Ronne-Engstrom E, Piper I, Nilsson P, Andrews P, Enblad P (2005). Pressure reactivity as a guide in the treatment of cerebral perfusion pressure in patients with brain trauma. J Neurosurg.

[20] Hutchinson PJ, Jalloh I, Helmy A, Carpenter KL, Rostami E, Bellander BM, Boutelle MG, Chen JW, Claassen J, Dahyot-Fizelier C, Enblad P, Gallagher CN, Helbok R, Hillered L, Le Roux PD, Magnoni S, Mangat HS, Menon DK, Nordstrom CH, O'Phelan KH, Oddo M, Perez Barcena J, Robertson C, Ronne-Engstrom E, Sahuquillo J, Smith M, Stocchetti N, Belli A, Carpenter TA, Coles JP, Czosnyka M, Dizdar N, Goodman JC, Gupta AK, Nielsen TH, Marklund N, Montcriol A, O'Connell MT, Poca MA, Sarrafzadeh A, Shannon RJ, Skjoth-Rasmussen J, Smielewski P, Stover JF, Timofeev I, Vespa P, Zavala E, Ungerstedt U (2015). Consensus statement from the 2014 international microdialysis forum. Intensive Care Med.

[21] Hutchinson PJ, Kolias AG, Czosnyka M, Kirkpatrick PJ, Pickard JD, Menon DK (2013). Intracranial pressure monitoring in severe traumatic brain injury. BMJ.

[22] Hyder AA, Wunderlich CA, Puvanachandra P, Gururaj G, Kobusingye OC (2007). The impact of traumatic brain injuries: a global perspective. NeuroRehabilitation.

[23] Khellaf A, Garcia NM, Tajsic T, Alam A, Stovell MG, Killen MJ, Howe DJ, Guilfoyle MR, Jalloh I, Timofeev I, Murphy MP, Carpenter TA, Menon DK, Ercole A, Hutchinson PJ, Carpenter KL, Thelin EP, Helmy A (2022). Focally administered succinate improves cerebral metabolism in traumatic brain injury patients with mitochondrial dysfunction. J Cereb Blood Flow Metab.

[24] Khellaf A, Khan DZ, Helmy A (2019). Recent advances in traumatic brain injury. J Neurol.

[25] Mathieu F, Khellaf A, Ku JC, Donnelly J, Thelin EP, Zeiler FA (2020). Continuous near-infrared spectroscopy monitoring in adult traumatic brain injury: a systematic review. J Neurosurg Anesthesiol.

[26] McCredie VA, Alali AS, Scales DC, Rubenfeld GD, Cuthbertson BH, Nathens AB (2018). Impact of ICU structure and processes of care on outcomes after severe traumatic brain injury: a multicenter cohort study. Crit Care Med.

[27] McIlvoy L (2004). Comparison of brain temperature to core temperature: a review of the literature. J Neurosci Nurs.

[28] Menon DK, Ercole A (2017). Critical care management of traumatic brain injury. Handb Clin Neurol.

[29] Mokri B (2001). The Monro-Kellie hypothesis: applications in CSF volume depletion. Neurology.

[30] Nelson DW, Thornquist B, MacCallum RM, Nystrom H, Holst A, Rudehill A, Wanecek M, Bellander BM, Weitzberg E (2011). Analyses of cerebral microdialysis in patients with traumatic brain injury: relations to intracranial pressure, cerebral perfusion pressure and catheter placement. BMC Med.

[31] Nielsen TH, Bindslev TT, Pedersen SM, Toft P, Olsen NV, Nordstrom CH (2013). Cerebral energy metabolism during induced mitochondrial dysfunction. Acta Anaesthesiol Scand.

[32] Nordstrom CH, Nielsen TH, Schalen W, Reinstrup P, Ungerstedt U (2016). Biochemical indications of cerebral ischaemia and mitochondrial dysfunction in severe brain trauma analysed with regard to type of lesion. Acta Neurochir (Wien).

[33] Okonkwo DO, Shutter LA, Moore C, Temkin NR, Puccio AM, Madden CJ, Andaluz N, Chesnut RM, Bullock MR, Grant GA, McGregor J, Weaver M, Jallo J, LeRoux PD, Moberg D, Barber J, Lazaridis C, Diaz-Arrastia RR (2017). Brain oxygen optimization in severe traumatic brain injury phase-II: a phase II randomized trial. Crit Care Med.

[34] Payen JF, Richard M, Francony G, Audibert G, Barbier EL, Bruder N, Dahyot-Fizelier C, Geeraerts T, Gergele L, Puybasset L, Vigue B, Skaare K, Bosson JL, Bouzat P (2020). Comparison of strategies for monitoring and treating patients at the early phase of severe traumatic brain injury: the multicentre randomised controlled OXY-TC trial study protocol. BMJ Open.

[35] Pease M, Nwachuku E, Goldschmidt E, Elmer J, Okonkwo DO (2022). Complications from multimodal monitoring do not affect long-term outcomes in severe traumatic brain injury. World Neurosurg.

[36] Ponce LL, Pillai S, Cruz J, Li X, Julia H, Gopinath S, Robertson CS (2012). Position of probe determines prognostic information of brain tissue PO2 in severe traumatic brain injury. Neurosurgery.

[37] Raabe A, Kopetsch O, Woszczyk A, Lang J, Gerlach R, Zimmermann M, Seifert V (2004). S-100B protein as a serum marker of secondary neurological complications in neurocritical care patients. Neurol Res.

[38] Raj R, Luostarinen T, Pursiainen E, Posti JP, Takala RSK, Bendel S, Konttila T, Korja M (2019). Machine learning-based dynamic mortality prediction after traumatic brain injury. Sci Rep.

[39] Reinstrup P, Unnerback M, Marklund N, Schalen W, Arrocha JC, Bloomfield EL, Sadegh V, Hesselgard K (2019). Best zero level for external ICP transducer. Acta Neurochir (Wien).

[40] Robba C, Cardim D, Tajsic T, Pietersen J, Bulman M, Rasulo F, Bertuetti R, Donnelly J, Xiuyun L, Czosnyka Z, Cabeleira M, Smielewski P, Matta B, Bertuccio A, Czosnyka M (2018). Non-invasive intracranial pressure assessment in brain injured patients using ultrasound-based methods. Acta Neurochir Suppl.

[41] Robba C, Donnelly J, Cardim D, Tajsic T, Cabeleira M, Citerio G, Pelosi P, Smielewski P, Hutchinson P, Menon DK, Czosnyka M (2019). Optic nerve sheath diameter ultrasonography at admission as a predictor of intracranial hypertension in traumatic brain injured patients: a prospective observational study. J Neurosurg.

[42] Romner B, Grande PO (2013). Traumatic brain injury: intracranial pressure monitoring in traumatic brain injury. Nat Rev Neurol.

[43] Ross MJ, McLellan SA, Andrews PJD (2010). Depth of intraparenchymal brain monitoring devices in neurosurgical intensive care. J Intensive Care Soc.

[44] Skrifvars MB, Sekhon M, Aneman EA (2021). Monitoring and modifying brain oxygenation in patients at risk of hypoxic ischaemic brain injury after cardiac arrest. Crit Care.

[45] Sorrentino E, Diedler J, Kasprowicz M, Budohoski KP, Haubrich C, Smielewski P, Outtrim JG, Manktelow A, Hutchinson PJ, Pickard JD, Menon DK, Czosnyka M (2012). Critical thresholds for cerebrovascular reactivity after traumatic brain injury. Neurocrit Care.

[46] Stein SC, Georgoff P, Meghan S, Mirza KL, El Falaky OM (2010). Relationship of aggressive monitoring and treatment to improved outcomes in severe traumatic brain injury. J Neurosurg.

[47] Steiner LA, Czosnyka M, Piechnik SK, Smielewski P, Chatfield D, Menon DK, Pickard JD (2002). Continuous monitoring of cerebrovascular pressure reactivity allows determination of optimal cerebral perfusion pressure in patients with traumatic brain injury. Crit Care Med.

[48] Tas J, Beqiri E, van Kaam RC, Czosnyka M, Donnelly J, Haeren RH, van der Horst ICC, Hutchinson PJ, van Kuijk SMJ, Liberti AL, Menon DK, Hoedemaekers CWE, Depreitere B, Smielewski P, Meyfroidt G, Ercole A, Aries MJH (2021). Targeting autoregulation-guided cerebral perfusion pressure after traumatic brain injury (COGiTATE): a feasibility randomized controlled clinical trial. J Neurotrauma.

[49] Tavakoli S, Peitz G, Ares W, Hafeez S, Grandhi R (2017). Complications of invasive intracranial pressure monitoring devices in neurocritical care. Neurosurg Focus.

[50] Teasdale G, Jennett B (1974). Assessment of coma and impaired consciousness. A practical scale. Lancet.

[51] Thelin EP, Carpenter KL, Hutchinson PJ, Helmy A (2017). Microdialysis monitoring in clinical traumatic brain injury and its role in neuroprotective drug development. AAPS J.

[52] Thelin EP, Nelson DW, Bellander BM (2014). Secondary peaks of S100B in serum relate to subsequent radiological pathology in traumatic brain injury. Neurocrit Care.

[53] Thelin EP, Nelson DW, Bellander BM (2017). A review of the clinical utility of serum S100B protein levels in the assessment of traumatic brain injury. Acta Neurochir (Wien).

[54] Thelin EP, Nelson DW, Ghatan PH, Bellander BM (2014). Microdialysis monitoring of CSF parameters in severe traumatic brain injury patients: a novel approach. Front Neurol.

[55] Thelin EP, Zeiler FA, Ercole A, Mondello S, Buki A, Bellander BM, Helmy A, Menon DK, Nelson DW (2017). Serial sampling of serum protein biomarkers for monitoring human traumatic brain injury dynamics: a systematic review. Front Neurol.

[56] Timofeev I, Carpenter KL, Nortje J, Al-Rawi PG, O'Connell MT, Czosnyka M, Smielewski P, Pickard JD, Menon DK, Kirkpatrick PJ, Gupta AK, Hutchinson PJ (2011). Cerebral extracellular chemistry and outcome following traumatic brain injury: a microdialysis study of 223 patients. Brain.

[57] Unden J, Ingebrigtsen T, Romner B, Scandinavian Neurotrauma C (2013). Scandinavian guidelines for initial management of minimal, mild and moderate head injuries in adults: an evidence and consensus-based update. BMC Med.

[58] Volovici V, Pisica D, Gravesteijn BY, Dirven CMF, Steyerberg EW, Ercole A, Stocchetti N, Nelson D, Menon DK, Citerio G, van der Jagt M, Maas AIR, Haitsma IK, Lingsma HF, Center-Tbi investigators pftICUs (2022). Comparative effectiveness of intracranial hypertension management guided by ventricular versus intraparenchymal pressure monitoring: a CENTER-TBI study. Acta Neurochir (Wien).

[59] Wheble JL, Menon DK (2016). TBI-the most complex disease in the most complex organ: the CENTER-TBI trial-a commentary. J R Army Med Corps.

[60] Zeiler FA, Ercole A, Beqiri E, Cabeleira M, Aries M, Zoerle T, Carbonara M, Stocchetti N, Smielewski P, Czosnyka M, Menon DK, Participants C-THRIS-S, Investigators (2019). Cerebrovascular reactivity is not associated with therapeutic intensity in adult traumatic brain injury: a CENTER-TBI analysis. Acta Neurochir (Wien).

[61] Zeiler FA, Iturria-Medina Y, Thelin EP, Gomez A, Shankar JJ, Ko JH, Figley CR, Wright GEB, Anderson CM (2021). Integrative neuroinformatics for precision prognostication and personalized therapeutics in moderate and severe traumatic brain injury. Front Neurol.

[62] Zeiler FA, Smielewski P (2018). Application of robotic transcranial Doppler for extended duration recording in moderate/severe traumatic brain injury: first experiences. Crit Ultrasound J.

[63] Zeiler FA, Thelin EP, Helmy A, Czosnyka M, Hutchinson PJA, Menon DK (2017). A systematic review of cerebral microdialysis and outcomes in TBI: relationships to patient functional outcome, neurophysiologic measures, and tissue outcome. Acta Neurochir (Wien).

[64] Ziegler D, Cravens G, Poche G, Gandhi R, Tellez M (2017). Use of transcranial Doppler in patients with severe traumatic brain injuries. J Neurotrauma.

